# Cigarette Smoke Reduces Fatty Acid Catabolism, Leading to Apoptosis in Lung Endothelial Cells: Implication for Pathogenesis of COPD

**DOI:** 10.3389/fphar.2019.00941

**Published:** 2019-08-29

**Authors:** Jiannan Gong, Hui Zhao, Tanzhen Liu, Lifang Li, Erjing Cheng, Shuyin Zhi, Lufei Kong, Hong-Wei Yao, Jianqiang Li

**Affiliations:** Department of Respiratory and Critical Care Medicine, The Second Hospital of Shanxi Medical University, Taiyuan, China

**Keywords:** chronic obstructive pulmonary disease, endothelial cells, metabolism, ceramide, apoptosis

## Abstract

Endothelial cell (EC) apoptosis contributes to cigarette smoke (CS)-induced pulmonary emphysema. Metabolism of glucose, glutamine, and fatty acid is dysregulated in patients with chronic obstructive pulmonary disease (COPD). Whether CS causes metabolic dysregulation in ECs leading to development of COPD remains elusive. We hypothesized that CS alters metabolism, resulting in apoptosis in lung ECs. To test this hypothesis, we treated primary mouse pulmonary microvascular ECs (PMVECs) with CS extract (CSE) and employed PMVECs from healthy subjects and COPD patients. We found that mitochondrial respiration was reduced in CSE-treated PMVECs and in PMVECs from COPD patients. Specifically, oxidation of fatty acids (FAO) was reduced in these cells, which linked to reduced carnitine palmitoyltransferase 1a (Cpt1a), an essential enzyme for carnitine shuttle. CSE-induced apoptosis was further increased when cells were treated with a specific Cpt1 inhibitor etomoxir or transfected with Cpt1a siRNA. L-Carnitine treatment augmented FAO but attenuated CSE-induced apoptosis by upregulating Cpt1a. CSE treatment increased palmitate-derived ceramide synthesis, which was reduced by L-carnitine. Although CSE treatment increased glycolysis, inhibiting glycolysis with 2-deoxy-d-glucose had no effects on CSE-mediated apoptosis in lung ECs. Conclusively, FAO reduction increases ceramide and apoptosis in lung ECs treated with CSE, which may contribute to the pathogenesis of COPD/emphysema.

## Introduction

Chronic obstructive pulmonary disease (COPD) is a progressive, obstructive disease of the lungs, and it becomes the third leading cause of death worldwide (Rennard and Drummond, 2015). The pathology of this disease is characterized by abnormal inflammatory responses, mucus hypersecretion, airway obstruction and remodeling, as well as alveolar destruction (Mirza et al., 2018). Cigarette smoke (CS) is a major risk factor for the development of COPD, which accounts for at least 75% of COPD deaths. CS contains thousands of chemicals, which contact with lung epithelial cells and damage them directly. This leads to cascade responses, including oxidative stress, protease/antiprotease imbalance, inflammation, apoptosis, and senescence, thereby causing lung injury (Yao and Rahman, 2011; Yue and Yao, 2016). In addition to the epithelial cells, endothelial cells (ECs) are also effector cells during CS-induced emphysema, as the volatile components in CS, such as aldehyde and acrolein, can directly enter into the blood vessels, causing EC injury (Kim et al., 2016; Voelkel, 2018). In fact, EC apoptosis is one of the contributing factors to the development of pulmonary emphysema (Petrache et al., 2005; Giordano et al., 2008; Garcia-Lucio et al., 2018). However, the mechanisms underlying CS-induced EC apoptosis are not fully understood.

Previous studies have shown that glucose, fatty acids, and glutamine metabolism are abnormal in patients with COPD (Kao et al., 2012; Wang et al., 2013; Telenga et al., 2014; Cruickshank-Quinn et al., 2018). These studies employed metabolomics, lipidomics, and metabolic flux to determine fuel metabolism using sputum, blood, and urine samples from patients with COPD.

These approaches provide advanced technology to detect a complete picture of the metabolic phenotype from COPD patients. For example, glucose production, clearance, and oxidation, and rate of glycolysis were increased in patients with COPD compared with control subjects. However, there is no change in the rate of nonoxidative disposal of pyruvate or serum lactate between healthy controls and COPD patients. (Kao et al., 2012). Lipid metabolism was dysregulated during the onset of COPD (Jiang et al., 2017). Glycerophospholipid and sphingolipid metabolism were associated with worse airflow obstruction, lung function decline, and COPD exacerbations (Cruickshank-Quinn et al., 2018). Compared to healthy controls, COPD patients displayed decreased lipoprotein and amino acids in serum and urine (Wang et al., 2013), including branched-chain amino acids, and increased glycerolphosphocholine in serum. Nevertheless, the cell-specific metabolic alterations in COPD remain elusive. CS exposure has been shown to reduce glycolysis in type II cells (Agarwal et al., 2014). Furthermore, acute CS exposure was shown to induce a switch from glucose to lipid as the main energy source and increase FAO in distal lung epithelial cells. It is unclear whether CS modulates metabolism in lung ECs. In this study, we hypothesize that CS alters metabolism in lung ECs, thereby causing apoptosis. Mitochondrion is a key organelle, which links to metabolism with apoptosis. Therefore, we tested this hypothesis to determine mitochondrial respiration and fuel utilization as well as its association with apoptosis in lung ECs treated with CS extract (CSE).

## Materials and Methods

### Cell Culture

Human pulmonary microvascular ECs (PMVECs) from healthy subjects and COPD patients with smoking history were obtained from Lonza (Walkersville, MD) and Cell Biologics (Chicago, IL). Cells were cultured in complete microvascular EC growth medium supplemented with the SingleQuots (Lonza). Culture medium was changed every 48 h. Cells (less than passage 6) with 70–80% confluency were used for experiments. Cells from each group were pooled for further treatment and analysis.

### Mouse PMVEC Isolation

Primary PMVECs were isolated as described previously with modifications (Suresh et al., 2017; Yao et al., 2019). C57BL/6J mice (8–10 weeks old, both male and female) were anesthetized, and lungs were removed. A mouse lung dissociation kit (Miltenyi Biotec) was used to enzymatically digest lungs. After removal of CD45^+^ cells, CD45^−^ cells were collected, washed, and incubated with CD31-conjugated beads (Invitrogen). CD31^+^ cells were enriched using a MACS column and magnetic field. The freshly isolated cells were considered as passage 0, which cultured in dish coated human fibronectin (30 µg/ml). Cells that were less than passage 5 were used for experiments. All animals were housed in accordance with the Guide for the Care and Use of Laboratory Animals. The animal study was approved by the Animal Care and Ethics Committee of the Second Hospital of Shanxi Medical School (CMTT#: 2013012) in accordance with international standards.

### CSE Preparation

Smoking from a Kentucky research 3R4F cigarette was bubbled into 10 ml of culture medium by a negative pressure pump at a speed of 1 min per cigarette. The optical density of this aqueous extract of smoking was measured at a wave length of 320 nm and adjusted into 1, which was considered as 10% CSE based on previous reports (Caito et al., 2010; Cui et al., 2018). The CSE was filtered through a 0.2-mm sterile filter to remove bacteria. Before each experiment, the CSE was freshly prepared so as to avoid the breakdown of aqueous substances and evaporation of volatile components. Control medium was prepared using air rather than smoking with the same procedure.

### Cell Treatment and Transfection

Cells were treated with L-carnitine (1 mM), etomoxir (10 μM), myriocin (100 nM), or palmitate–bovine serum albumin (BSA) (50 μM) based on preliminary studies and publications (Yao et al., 2019). Mouse PMVECs cells were cultured onto a six-well plate with the density of 1 × 10^6^/well. Carnitine palmitoyltransferase 1a (Cpt1a) siRNA (Cat#: AM16708, IDs: 161677 and 161678, Thermo Fisher Scientific) at 50 nM was added with the Lipofectamine 2000 (Invitrogen) for 24 h to reduce endogenous Cpt1a expression. In each well, the volume of siRNA and Lipofectamine 2000 was 5 and 5 μl, respectively.

### Viability Assay

Cell viability was assessed after the addition of 3-(4,5-dimethylthiazol-2-yl)-2,5-diphenyl tetrazolium bromide (MTT, 4 mg/ml) for the final 5 h. MTT incorporation was measured at a 570-nm wavelength by a microplate absorbance reader (Biorad-550).

### Measurement of Oxygen Consumption Rate and Substrate Oxidation

Real-time oxygen consumption rate (OCR) was recorded using the Seahorse XF-24 Analyzer (Seahorse Bioscience). Cells at 15,000 cells per well were seeded in a Seahorse culture microplate. OCR was determined after sequential injections with oligomycin (1 μM), carbonyl cyanide p-trifluoromethoxyphenylhydrazone (FCCP, 0.5 μM), and rotenone/antimycin A (0.5 μM) according to the manufacturer’s manual. The numbers of cells in well was used to normalize the OCR values. Mitochondrial oxidation of fatty acids, glucose, and glutamine were determined by the Seahorse Analyzer following the instruction of the Seahorse XF Mito Fuel Flex Test kit (Pike Winer and Wu, 2014). In brief, etomoxir (4 μM), 2-cyano-3-(1-phenyl-1H-indol-3-yl)-2-propenoic acid (UK5099, 2 μM), and bis-2-(5-phenylacetamido-1,3,4-thiadiazol-2-yl)ethyl sulfide (BPTES, 3 μM) were sequentially injected from the cartridge plate during the OCR measurement. Etomoxir, UK5099, and BPTES are specific inhibitors of fatty acid, glucose, and glutamine oxidation, respectively. The substrate oxidation utilization was calculated as the percentage of specific pathway utilization to the whole OCR levels.

### Mass Spectrometry Analysis

To directly measure fatty acid oxidation (FAO), cells were incubated with medium supplemented with 100 µM [U-^13^C] palmitate or palmitate conjugated to BSA for 6 h (Yao et al., 2016). Palmitate/BSA conjugation was performed when 1 mM sodium palmitate (Sigma) or 1 mM potassium-U-13C palmitate (Cambridge Isotopes) was conjugated with 0.17 mM fatty acid free-BSA (Sigma) in 150 mM NaCl solution at 37°C for 1 h. We extracted cells as previously described (Vaskovsky et al., 1975; Ivanisevic et al., 2013). The Agilent 6530 Q-TOF was used to identify and analyze levels of citrate, α-ketoglutarate, fumarate, malate, and ceramide, which was confirmed by comparing retention times and tandem mass spectrometry data with standard compounds. The results were corrected for naturally occurring ^13^C impurity of the tracers.

### RNA Extraction and Real-Time Polymerase Chain Reaction

RNeasy miniprep kit (Qiagen) was used to purify DNA after extraction by the TRIzol reagent. RNA concentrations were measured by the NanoDrop spectrophotometer. We used 500 ng of total RNAs for reverse transcription with the Taqman^®^ Reverse Transcription Reagents (ThermoFisher Scientific). Real-time polymerase chain reaction (PCR) was performed using 1 μl of cDNA by the 7300 Real-Time PCR System (Applied Biosystems). All TaqMan gene probes were purchased from the Thermo Fisher Scientific. Mm01231183, Mm00487191, and Mm00463970 are the catalog numbers for Cpt1a, Cpt1b, and Cpt1c probes, respectively. 18s rRNA was used as a housekeeping gene for normalization. RNA levels were calculated by the comparative 2^−ΔΔCt^ method.

### Western Blot

Cells were lyzed with 0.3 ml of radioimmunoprecipitation assay buffer (50 mmol/l Tris–HCl, 150 mmol/l NaCl, 1 mmol/l EDTA, 0.25% deoxycholate, 1 mmol/l NaF, 0.5 mmol/l Na_3_VO_4_, 1 mg/l aprotinin, 1 mg/l leupeptin, and 0.5 mmol/l phenylmethylsulfonyl fluoride). We kept the lysates on ice for 30 min and then passed them three times through a 22-gaugue needle to disassociate the DNA. Whole cell lysates were centrifuged at 10,000 rpm in an Eppendorf tube for 5 min in 4°C, and the supernatant was collected. A bicinchoninic acid (BSA) Protein Assay kit (Thermo Scientific, Rockford, IL, USA) was used to measure protein levels in supernatants. Samples (10–20 μg proteins) were separated on a 10% sodium dodecyl sulfate–polyacrylamide gel electrophoresis gel (Invitrogen), and separated proteins were electroblotted onto nitrocellulose membranes. The blots were blocked for 1 h at room temperature with 5% milk, and we probed them with 1:1,000 diluted antibody against Cpt1a (Cat#: ab128568, Cell Signaling). The abundance of protein was determined using secondary antibodies with 1:10,000 dilutions linked to horseradish peroxidase. The ChemiDoc^™^ Imaging System was used to detect the band with the enhanced chemiluminescence method. β-Actin was used as a housekeeping control.

### Flow Cytometric Assay for Annexin V-Positive Cells

Flow cytometry was used to measure Annexin V-positive cells that are considered as apoptotic cells. Briefly, cells were collected using the TrypLE (Thermo Fisher Scientific). After washed with cold phosphate buffered saline, Annexin V binding buffer (300 µl) was added to resuspend cells. A total 0.1 × 10^6^ cells were collected and incubated with 100 μl Annexin V binding buffer along with 5 μl of fluorescein isothiocyanate conjugated Annexin V (Life Technologies, USA) and 5 μl of propidium iodide (Life Technologies, USA) for 20 min at room temperature. Finally, we added Annexin V binding buffer (500 μl) and mixed gently. FC-500 (Beckman Coulter) was used to detect the Annexin V-positive cells with total 20,000 events analyzed.

### Statistical Analysis

Experiments were performed at least three biological replicates with total *n* = 5–6 times of measurements. Data are expressed as mean ± SEM. One-way analysis of variance (ANOVA) was used to determine whether there are any statistical significance between the means of groups. The Student–Newman–Keuls (SNK) test was used to examine which specific groups of means were statistically different. Statistical significance was considered when *P* < 0.05.

## Results

### Oxidative Phosphorylation Was Reduced in CSE-Treated Mouse PMVECs and in PMVECs From COPD Patients

**Figure 1:**
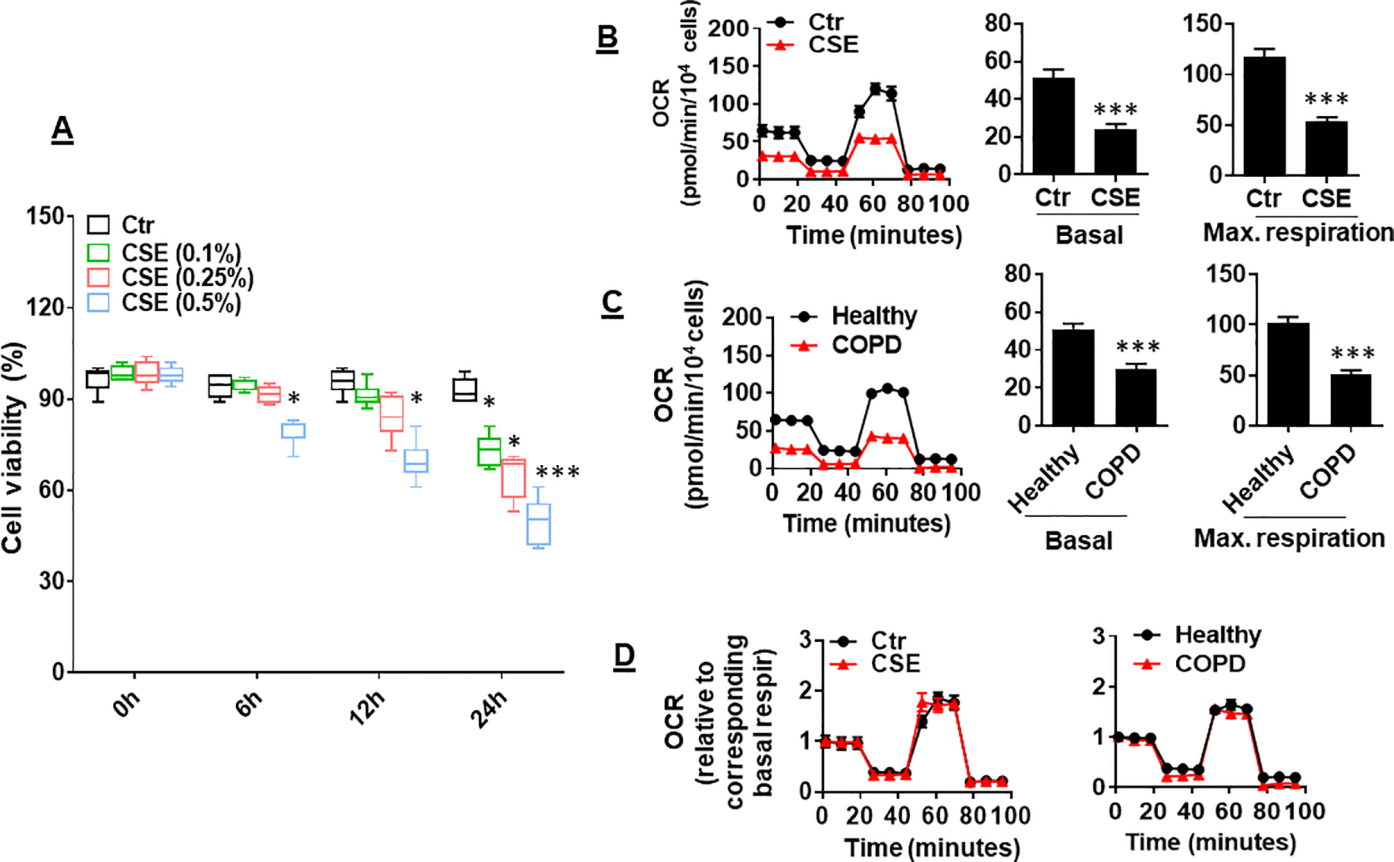
Mitochondrial respiration was reduced in cigarette smoke (CSE)-treated mouse primary mouse pulmonary microvascular endothelial cells (PMVECs) and in PMVECS from chronic obstructive pulmonary disease (COPD) patients. **(A)** Mouse PMVECs were treated with control and CSE (0.1–0.5%) for 6–24 h. 3-(4,5-Dimethylthiazol-2-yl)-2,5-diphenyl tetrazolium bromide (MTT) assay was performed to measure cell viability. **(B)** Mouse PMVECs were exposed to CSE (0.25%, 12 h), and then, oxygen consumption rate (OCR) was measured by the Seahorse XF24 Analyzer. Kinetic OCR response to oligomycin (1 μM), carbonyl cyanide p-trifluoromethoxyphenylhydrazone (FCCP, 0.5 µM), and rotenone/antimycin A (0.5 μM) was recorded. Basal and maximal respiration were normalized to the number of live cells. **(C)** PMVECs from healthy subjects and COPD patients were used to measure the OCR by the Seahorse XF24 Analyzer. **(D)** Kinetic changes of mitochondrial respiration after normalization with corresponding basal respiration. Data are expressed as mean ± SEM, *n* = 5–6. *P < 0.05, ***P < 0.001 vs. control (Ctr), or healthy group.

CS has been shown to reduce mitochondrial respiration in alveolar epithelial cells (Agarwal et al., 2014). It is unknown whether CS alters mitochondrial respiration in lung ECs. To answer this question, we determined the effect of CSE on mitochondrial respiration in primary mouse PMVECs. We first treated cells with CSE (0.1, 0.25, and 0.5%) for 6, 12, and 24 h and found that CSE reduced cell viability in a dose- and time-dependent manner (**Figure 1A**). Thus, we chose CSE (0.25%, 12 h), which may impair cell function (e.g., apoptosis) but not affect cell viability, for following experiments. Treatment with CSE (0.25%, 12 h) significantly reduced oxidative phosphorylation in mouse PMVECs, which was shown by the decreased basal and maximal respiration compared to control group (**Figure 1B**). Similarly, the OCR was decreased in PMVECs from COPD patients (**Figure 1C**). Once normalized to their corresponding basal respiration, we did not observe any significant changes in kinetic response of mitochondrial respiration between air and CSE groups (**Figure 1D**). This suggests that CS exposure results in a reduction in mitochondrial respiration, particularly in basal respiration.

### FAO Was Reduced in CSE-Treated Mouse PMVECs and in PMVECs From COPD Patients

**Figure 2:**
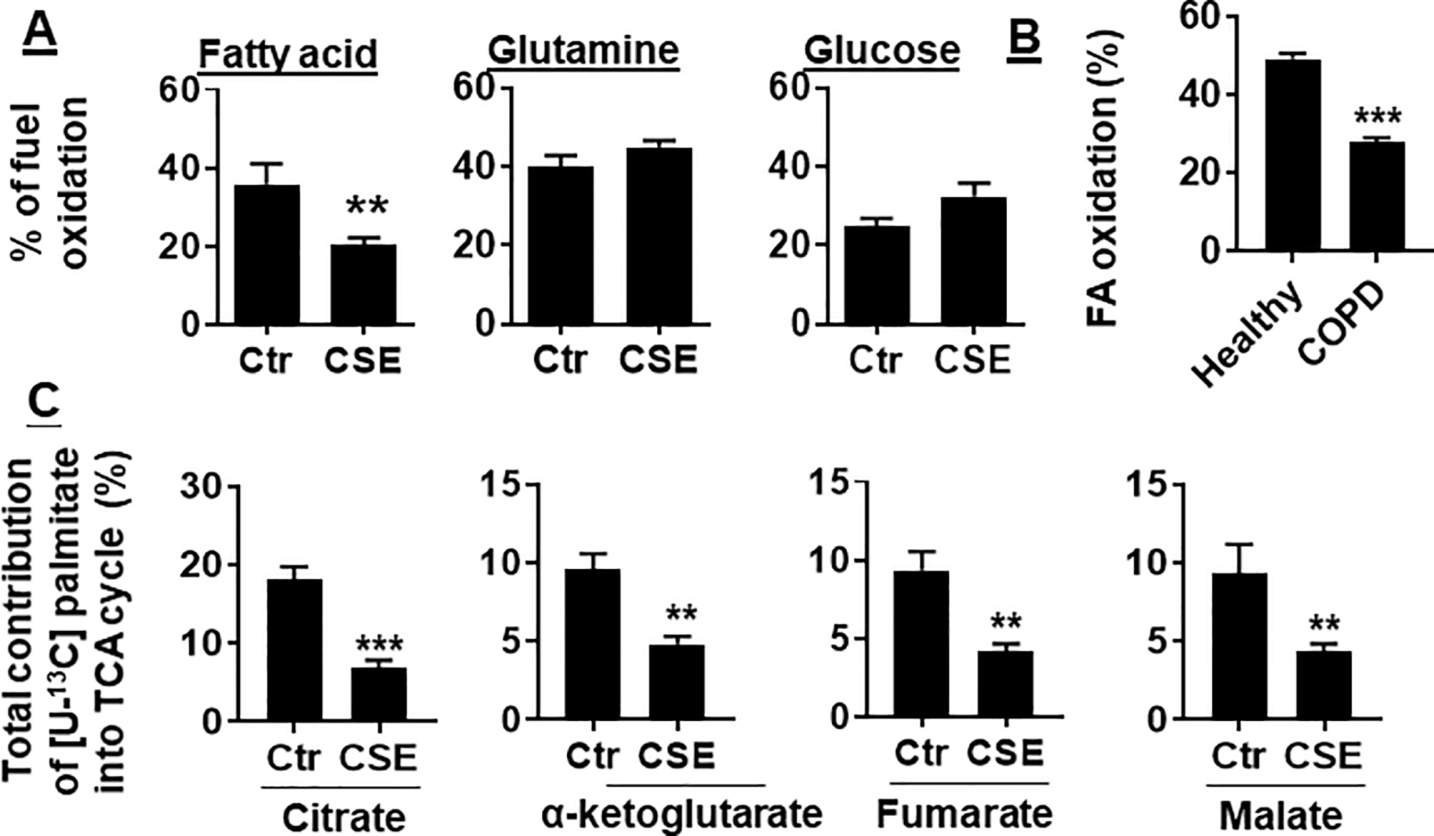
FAO was reduced in CSE-treated mouse PMVECs and in PMVECs from COPD patients. **(A)** Mouse PMVECs were exposed to CSE (0.25%) for 12 h. Fuel utilization was measured in cells by the Seahorse XF24 Analyzer when the pathway inhibitors: etomoxir (4 μM), BPTES (3 μM), and UK5099 (2 μM) were injected. The oxidation of FA, glutamine, and glucose was calculated as the difference of OCR between etomoxir, BPTES, or UK5099 and vehicle treatments, respectively. **(B)** FAO was measured in PMVECs from healthy subjects and COPD patients using the Seahorse XF24 Analyzer. **(C)** After treatment of mouse PMVECs with (0.25%, 12 h), U-^13^C palmitate labeled (50 μM) was added for 24 h. Levels of 13C-labeled citrate, α-ketoglutarate, fumarate, and malate were measured using mass spectrometry. Data are expressed as mean ± SEM, *n* = 5–6. **P < 0.01, ***P < 0.001 vs. control (Ctr) or healthy group.

To determine whether CS exposure alters substrate utilization by mitochondria, we determined the oxidation of glucose, glutamine, and long-chain FA in human PMVECs using the Mitochondrial Fuel Flex Test kit. We found that the percentage of FAO was reduced in human PMVECs treated with CSE (0.25%) for 12 h. There were no changes in glucose or glutamine oxidation in human PMVECs treated with CSE (0.25%) for 12 h (**Figure 2A**). Furthermore, the utilization of FAs was significantly reduced in PMVECs from COPD patient as compared to healthy subject (**Figure 2B**). To further directly determine the oxidation of FAs, we treated human PMVECs with U-^13^C-labeled palmitate and measured the levels of citrate, α-ketoglutarate, fumarate, and malate derived from exogenous palmitate. As shown in **Figure 2C**, the levels of palmitate-derived citrate, α-ketoglutarate, fumarate, and malate were significantly reduced in human PMVECs treated with CSE (0.25%, 12 h) compared to air control. Altogether, these results demonstrate that FAO is reduced in human PMVECs exposed to CS.

### Cpt1a Levels Was Reduced in CSE-Treated Mouse PMVECs and in PMVECs From COPD Patients

**Figure 3:**
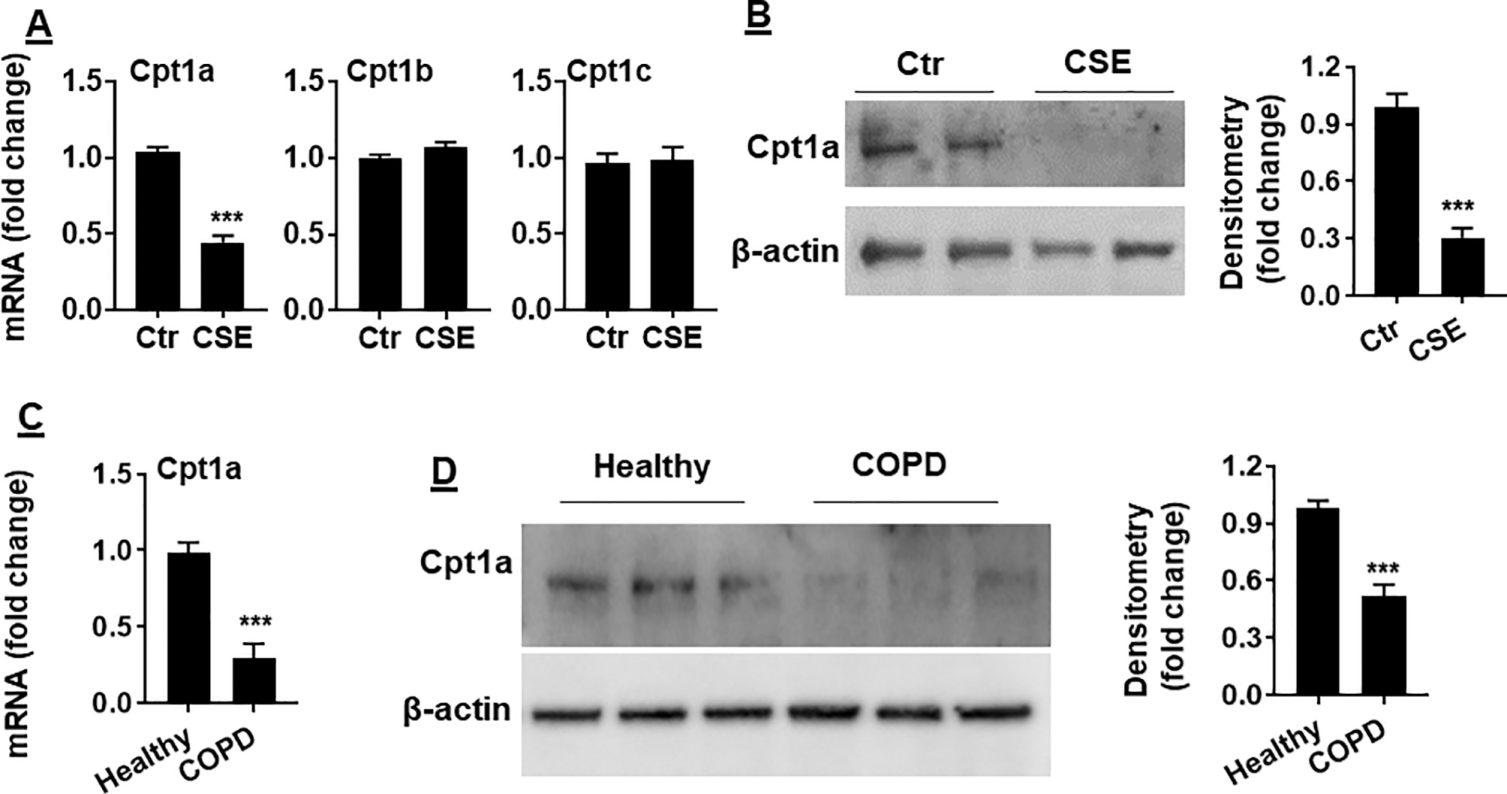
Cpt1a levels were reduced in CSE-treated mouse PMVECs and in PMVECs from COPD patients. **(A)** Mouse PMVECs were treated with CSE (0.25%, 12 h), RNA was extracted, and Cpt1a, Cpt1b, and Cpt1c mRNAs were measured by qRT-PCR. **(B)** Levels of Cpt1a protein were measured in mouse PMVECs treated with CSE by Western blot. **(C)** Cpt1a mRNA was measured by qRT-PCR in PMVECs from healthy subjects and COPD patients. **(D)** Levels of Cpt1a protein were measured in human PMVECs by Western blot. Data are expressed as mean ± SEM, *n* = 5–6. ***P < 0.001 vs. control (Ctr) or healthy group.

Carnitine shuttle transfers the acyl group of long-chain FAs into mitochondria, where Cpt1 is an essential enzyme for carnitine shuttle. Hence, we measured the mRNA levels of Cpt1 isoforms including Cpt 1a, Cpt 1b, and Cpt 1c. As shown in **Figure 3A**, Cpt1a mRNA was significantly reduced in mouse PMVECs treated with CSE. Similarly, CSE treatment decreased Cpt1a protein in mouse PMVECs (**Figure 3B**). The levels of Cpt1a mRNA and proteins were significantly reduced in PMVECs from patients with COPD when compared with healthy subjects (**Figures 3C, D**). These data suggest that CS-induced FAO reduction is due to the decreased levels of Cpt1a.

### FAO Augmentation Ameliorated CSE-Induced Apoptosis

**Figure 4:**
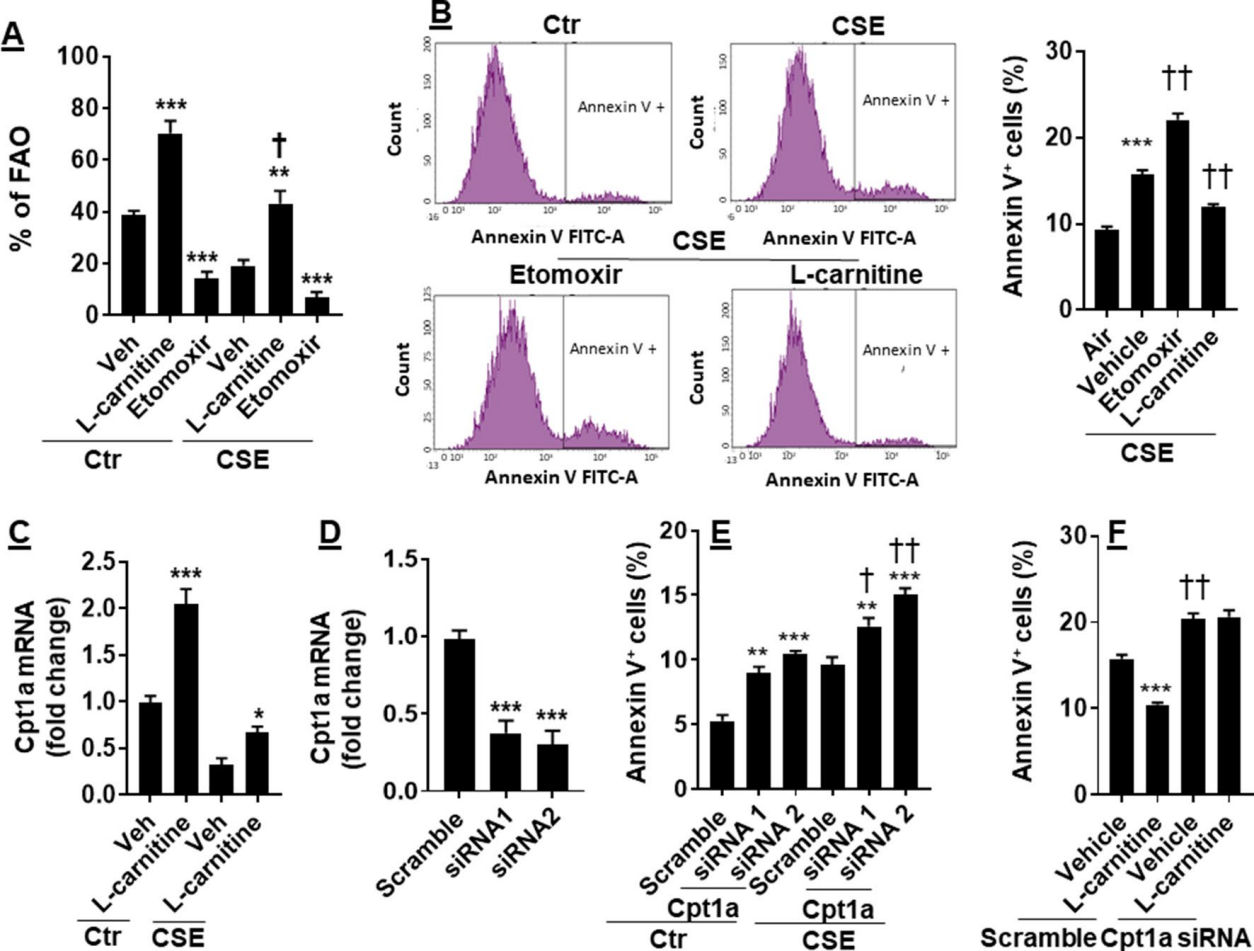
Enhancing FAO attenuated CSE-induced apoptosis. Mouse PMVECs were treated with L-carnitine (1 mM, 12 h) or etomoxir (10 μM, 12 h) in the absence or presence of CSE (0.25%, 12 h). **(A)** FAO was measured by the Seahorse Analyzer. **P < 0.01, ***P < 0.001 vs. vehicle (veh); ^†^*P* < 0.05 vs. Ctr/L-carnitine. **(B)** Annexin V^+^ cells were assessed by flow cytometry. ***P < 0.001 vs. air; ^††^*P* < 0.01 vs. CSE/vehicle. **(C)** Cpt1a mRNA was measured by qRT-PCR. *P < 0.05, ***P < 0.001 vs. vehicle (veh). **(D)** Mouse PMVECs were transfected with scramble or Cpt1a siRNA, and Cpt1a mRNA was measured by qRT-PCR. ***P < 0.001 vs. scramble siRNA. **(E)** Annexin V^+^ cells were assessed by flow cytometry in scramble or Cpt1a siRNA transfected cells in response to CSE (0.25%, 12 h) treatment. **P < 0.01, ***P < 0.001 vs. scramble siRNA; ^†^*P* < 0.05, ^††^ *P* < 0.01 vs. Ctr. **(F)** Cpt1a siRNA transfected cells were treated with L-carnitine in response to CSE (0.25%, 12 h) treatment. Annexin V^+^ cells were assessed by flow cytometry. ***P < 0.001 vs. vehicle (veh); ^††^*P* < 0.01 vs. scramble siRNA. Data are expressed as mean ± SEM, *n* = 5–6.

CS has been shown to cause apoptosis in lung ECs (Chen et al., 2012; Yang et al., 2015). It is not clear whether manipulation of FAO affects CS-induced apoptosis. To answer this question, we treated CSE (0.25%, 12 h)-exposed mouse PMVECs with L-carnitine (1 mM) or etomoxir (10 μM) for 12 h. As shown in **Figure 4A**, inhibition of Cpt1 by etomoxir reduced, whereas supplementing L-carnitine as a substrate of Cpt1 enhanced, the FAO in PMVECs in both control and CSE groups. The apoptosis determined by the Annexin V Apoptosis Detection kit was further increased in CSE-exposed PMVECs treated with etomoxir, and these effects were attenuated by L-carnitine (**Figure 4B**).

We next asked whether L-carnitine treatment alters Cpt1a expression. As expected, CSE treatment decreased Cpt1a gene expression (**Figure 4C**). L-Carnitine treatment increased expression of Cpt1a mRNA in both control and CSE groups (**Figure 4C**). We next silenced Cpt1a to determine whether this affects L-carnitine’s effect on apoptosis. Transfection of Cpt1a siRNA significantly reduced Cpt1a gene expression (**Figure 4D**). Knockdown of Cpt1a increased Annexin V-positive cells, which was further augmented by CSE treatment (**Figure 4E**). Furthermore, Cpt1a silencing abolished L-carnitine’s effect on apoptosis in response to CSE treatment (**Figure 4F**). All the results demonstrate that enhancing FAO attenuates CSE-induced apoptosis by upregulating Cpt1a in PMVECs.

### CSE Increased Ceramide Synthesis, Which Was Modulated by the FAO

**Figure 5:**
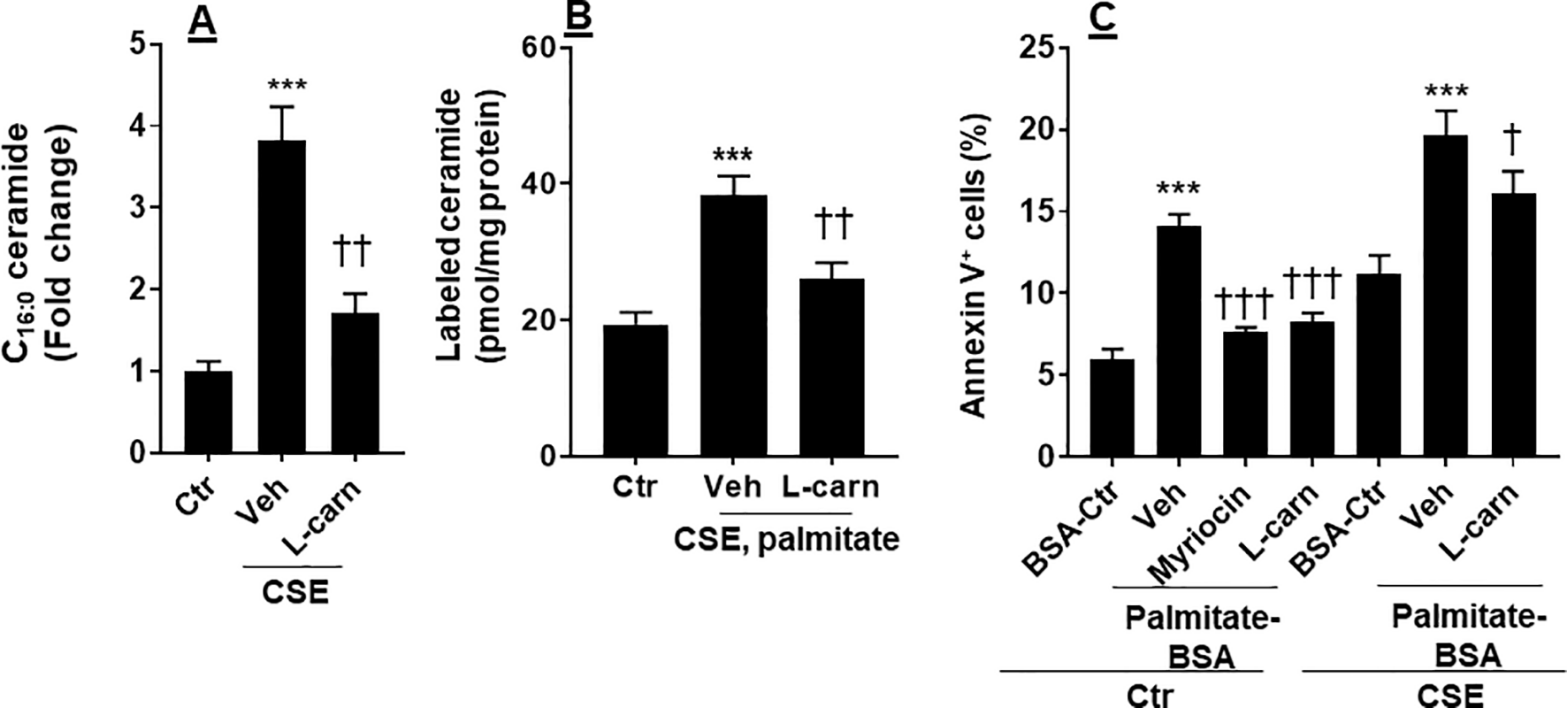
CSE increased palmitate-derived ceramide leading to apoptosis. **(A)** Mouse PMVECs were treated with CSE (0.25%, 12 h) in the absence or presence of L-carnitine (1 mM, 12 h) treatment. Ceramide were measured in cells by mass spectrometry. (B) After CSE (0.25%, 12 h) along with L-carnitine (1 mM, 12 h) treatment, U-^13^C palmitate (100 µM) was added for 6 h incubation. ^13^C-Ceramide was measured by mass spectrometry. **(C)** Mouse PMVECs were treated CSE (0.25%, 12 h) along with L-carnitine (1 mM) and myriocin (100 nM) for 12 h in the presence of palmitate–BSA or control BSA for 12 h. Annexin V-positive cells were measured by flow cytometry. Data are expressed as mean ± SEM, *n* = 5–6. ***P < 0.001 vs. air control (Ctr); ^†^ *P* < 0.05, ^††^ *P* < 0.01, ^†††^ *P* < 0.001 vs. CSE/vehicle or palmitate–BSA/vehicle (veh).

Ceramide is able to cause apoptosis in ECs (Paumen et al., 1997). We determined whether CSE increases ceramide synthesis, and this effect is modulated by the FAO. As shown in **Figure 5A**, CSE treatment increased the levels of C16:0 ceramide in PMVECs, which was reduced by L-carnitine (1 mM). Next, we treated human PMVECs with U-^13^C-palmitate (100 µM, 6 h) and determined palmitate-derived ceramide by mass spectrometry. CSE treatment increased ^13^C-labeled ceramides, and these effects were attenuated when PMVECs were treated with L-carnitine (**Figure 5B**). In addition, we treated cells with a specific inhibitor of *de novo* ceramide synthesis (myriocin, 100 nM, 12 h) and L-carnitine (1 mM, 12 h) in the presence of exogenous palmitate-BSA (50 µM, 12 h). As shown in **Figure 5C**, treatment with exogenous palmitate significantly augmented apoptosis as detected by the Annexin V Apoptosis Detection kit compared with control. Treatments with L-carnitine exhibited a similar effect to myriocin in reducing palmitate-induced apoptosis in PMVECs (**Figure 5C**). Altogether, these results suggest that CS increases the conversion from fatty acids to ceramide, leading to apoptosis, and these effects are inhibited by enhancing FAO.

### Glycolysis Was Increased by CS Exposure, Which Was Not Associated With Apoptosis

**Figure 6:**
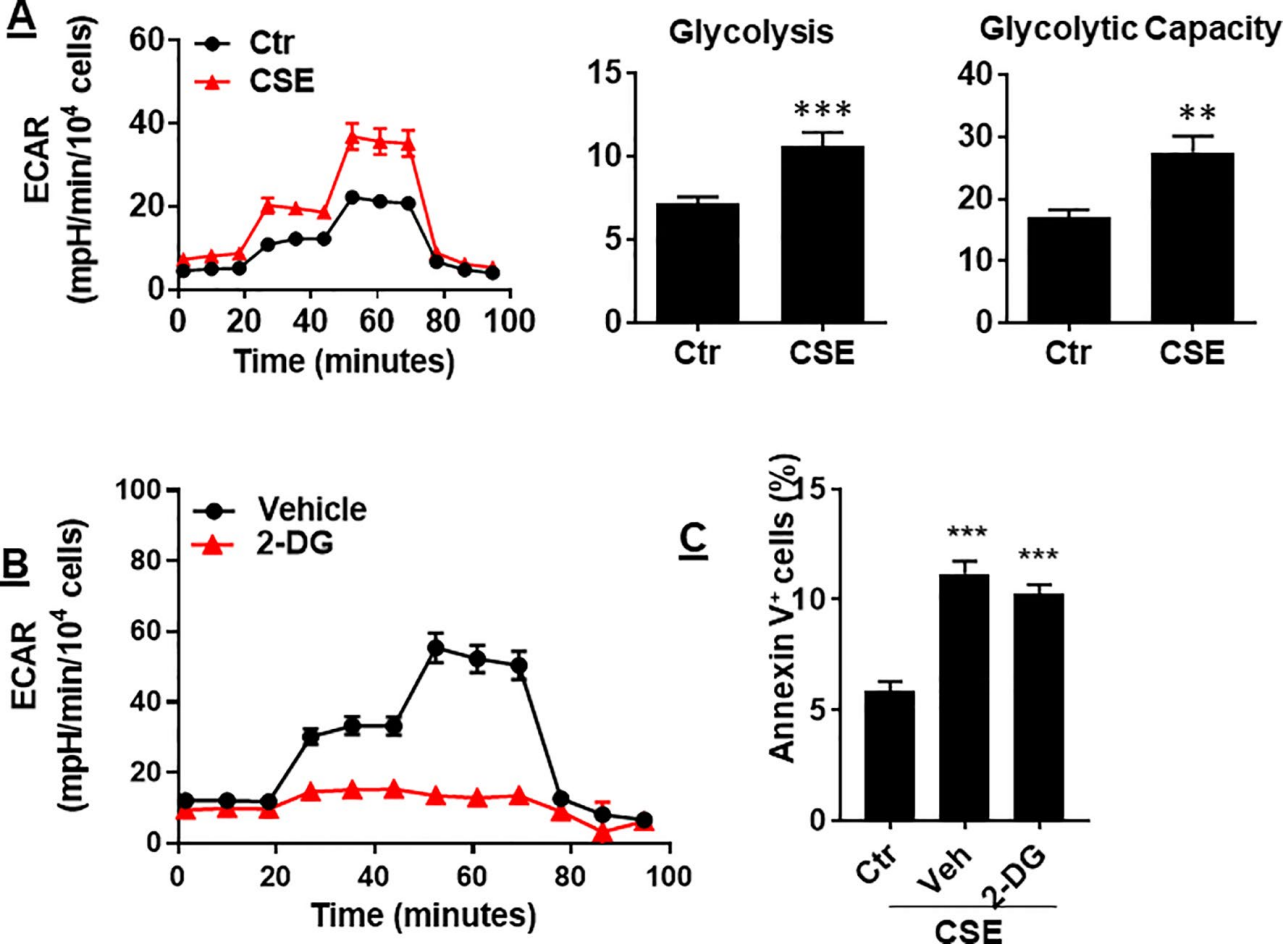
CSE increased glycolysis, which was not associated with apoptosis. **(A)** Extracellular acidification rate (ECAR) was measured by the Seahorse Analyzer in mouse PMVECs exposed to CSE (0.25%, 12 h). **(B)** Mouse PMVECs were treated with 2-DG (3 mM, 12 h), glycolysis was measured by the Seahorse Analyzer. **(C)** Mouse PMVECs were treated with 2-DG (3 mM, 12 h) in the presence of CSE (0.25%, 12 h). Annexin V^+^ cells were assessed by flow cytometry. Data are expressed as mean ± SEM, *n* = 3–6, **P < 0.01, ***P < 0.001 vs. air control (Ctr).

In ECs, glycolysis is the main metabolic pathway for generating bioenergetics. Thus, we determined whether CSE treatment alters glycolysis, and this associates with apoptosis. When mouse PMVECs were treated with CSE (0.25%, 12 h), glycolysis was increased as reflected by increased extracellular acidification rate (ECAR) (**Figure 6A**). Inhibition of glycolysis by 2-deoxy-D-glucose (3 mM, 12 h) had no effects on CSE-induced increase in Annexin V-positive cells (**Figure 6B**). The results suggest that CS-induced apoptosis in PMVECs is not associated with glycolysis.

## Discussion

**Figure 7:**
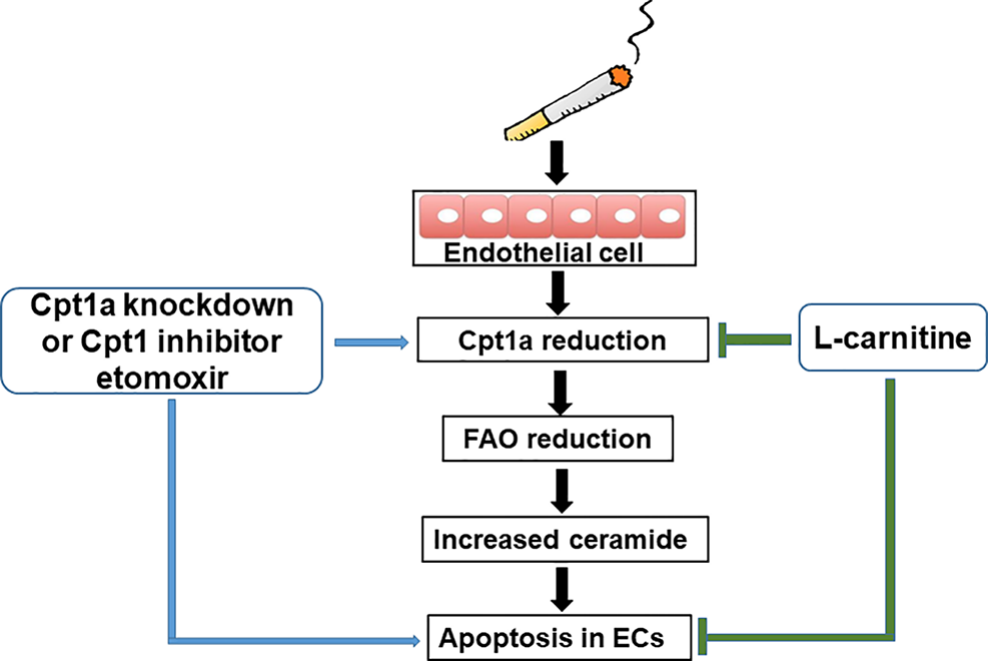
A schematic figure summarizing the present study. Cigarette smoke causes apoptosis by downregulating fatty acid oxidation and increasing ceramide synthesis in lung endothelial cells.

Previous studies have proven that COPD patients have problems with their metabolism (Paige et al., 2011; Kao et al., 2012; Wang et al., 2013). There are no reports studying metabolic reprogramming in CS-induced apoptosis in lung EC. Here, we showed that CSE treatment reduced mitochondrial respiration in lung ECs, which were consistent with the results of decreased mitochondrial respiration in lung ECs by acrolein (Lu et al., 2017). Specifically, we also showed that CSE decreased FAO in lung ECs. This was associated with increased ceramide synthesis and apoptosis. Enhancing FAO by L-carnitine reduced, where inhibiting FAO by etomoxir or genetic knockdown of Cpt1a further augmented, CSE-induced apoptosis in lung ECs (**Figure 7**). Hence, CSE treatment reduces FAO, leading to increased ceramide synthesis and apoptosis in lung ECs.

Compared to nonsmokers, primary basal stem/progenitor cells in the airways in healthy smoker had reduced metabolites in the tricarboxylic acid cycle, including acetyl-CoA, NADH, and FADH2 (Deeb et al., 2016). In contrast, an animal study has shown that CS exposure increased the levels of malate, citrate, and fumarate in rat lungs (Barupal et al., 2016). These studies suggest that CS may affect mitochondrial oxidative phosphorylation in a cell-specific manner. We found that CSE reduced mitochondrial respiration in lung ECs, which was determined by measuring the OCR *via* electron transport chain. A previous report has shown that CS blocked the mitochondrial respiratory chain in lung epithelial cells (Van Der Toorn et al., 2007). It is unknown whether CS impairs either the mitochondrial respiratory chain or tricarboxylic acid cycle, thereby reducing mitochondrial respiration in lung ECs. Further study is required to determine whether CS and it components impair oxidative phosphorylation in a dose- and time-dependent manner.

Cpt1 is a rate-limiting enzyme for carnitine shuttle during the FAO. There are a few studies showing that CS induced expression of Cpt1a, thereby enhancing FAO in lung epithelial cells and mouse lungs (Agarwal et al., 2014; Jiang et al., 2017). In contrast, FAO is impaired in alveolar epithelial cells during pathogen-induced acute lung injury (Cui et al., 2019). We found that CS exposure reduced Cpt1a levels and FAO in lung ECs. The discrepancies may be due to differences in epithelial cells, ECs, and lungs, as well as CS exposure durations. The limitation in our study is that we used commercial human PMVECs, which were not able to provide demographic information of subjects. Further study, using PMVECs from COPD patients with stratified demographic characteristics, including age, gender, smoking history, and disease severity, would reveal the effects of these features on Cpt1a and FAO during COPD progression.

We found that the levels of Cpt1b or Cpt1c were not detectable by Western blot in lung ECs (data not shown). This is in agreement with the report that Cpt1a is abundantly expressed, while Cpt1b or Cpt1c is expressed at very low levels in rat lungs (Ceccarelli et al., 2011). L-Carnitine is a metabolite critical for transporting long-chain fatty acids into the mitochondria for subsequent β-oxidation. It has been shown that L-carnitine is reduced in mouse lungs with emphysema (Conlon et al., 2016). As a substrate of Cpt1, L-carnitine could increase Cpt1 gene expression, protein levels and activity (Karlic et al., 2002; Xi et al., 2008). This is in agreement with our finding that L-carnitine treatment increased Cpt1a gene expression in lung ECs. This contributes to the protection of L-carnitine against CSE-induced apoptosis.

The levels of sphingolipids, including ceramide, were significantly increased in sputum from smokers with COPD compared to smokers without COPD (Telenga et al., 2014). C16-Ceramide accumulation could cause mitochondrial damage and necroptosis in lung epithelial cells (Mizumura et al., 2018). This is in agreement with our findings that CS exposure increased levels of ceramide in lung ECs. Ceramide causes apoptosis and emphysema of lung ECs (Petrache et al., 2005; Garcia-Lucio et al., 2018). In fact, reducing ceramide synthesis by myriocin reduced CSE-induced apoptosis in lung ECs. The mechanisms underlying CS-induced increase in ceramide are associated with reduced FAO; however, it remains elusive whether Cpt1a knockdown affects CSE-induced increase in ceramide. This was evidenced by the findings that enhancing FAO by L-carnitine reduced CS-induced ceramide synthesis and apoptosis in lung ECs. Whether FAO reduction also mediates necroptosis in lung ECs exposed to CS remains elusive. L-Carnitine administration attenuates elastase-induced pulmonary emphysema (Conlon et al., 2016). A clinical study has shown that carnitine can improve exercise tolerance and inspiratory muscle strength in COPD patients (Borghi-Silva et al., 2006). Altogether, these findings suggest that enhancing FAO by L-carnitine may be beneficial to COPD/emphysema.

A previous report has shown that glycolysis was reduced in in the CS-exposed alveolar type II cells (Agarwal et al., 2014). We found that CSE treatment increased glycolysis in lung ECs. It remains elusive whether these cells may attempt to restore glycolysis to generate ATP or lactate to feed the tricarboxylic acid cycle as a compensatory mechanism against reduced mitochondrial respiration for bioenergetics and cellular function (Hui et al., 2017). Inhibition of glycolysis with 2-deoxy-D-glucose had no significant effect on CSE-induced apoptosis. Hence, glycolysis is not involved in CSE-induced apoptosis in lung ECs.

In conclusion, CSE treatment reduced mitochondrial respiration and FAO, leading to apoptosis in lung ECs. The mechanisms underlying CSE-induced apoptosis is associated with increased ceramide synthesis in condition of insufficient FAO. L-Carnitine enhances FAO but attenuates CSE-induced ceramide levels and apoptosis by upregulating Cpt1a in lung ECs. Therefore, the reduction in FAO in lung ECs may contribute to pathogenesis of CS-induced COPD/emphysema.

## Data Availability

The raw data supporting the conclusions of this manuscript will be made available by the authors, without undue reservation, to any qualified researcher.

## Ethics Statement

The animal study was approved by the Animal Care and Ethics Committee of the second Hospital of Shanxi Medical School (CMTT#: 2013012) in accordance with international standard.

## Author Contributions

Conception and design: HWY and JL; data acquisition and analysis: JG, HZ, TL, LL, EC, SZ, and LK; data interpretation: JG, HWY, and JL; drafting the manuscript: JG; revising the manuscript: JG, HWY, and JL.

## Funding

This work was supported by the Education Department of Shanxi Province Foundation for Graduate Education Innovation (#2018BY068) and Health Commission of Shanxi Province Foundation for Higher Education Technology Innovation (#2017050).

## Conflict of Interest Statement

The authors declare that the research was conducted in the absence of any commercial or financial relationships that could be construed as a potential conflict of interest.

## Abbreviations

COPD, chronic obstructive pulmonary disease; Cpt, carnitine palmitoyltransferase; CS, cigarette smoke; CSE, cigarette smoke extract; ECAR, extracellular acidification rate; ECs, endothelial cells; FAO, fatty acid oxidation; PMVECs, lung microvascular endothelial cells; OCR, oxygen consumption rate.
